# Polymorphism in COX-2 modifies the inverse association between *Helicobacter pylori *seropositivity and esophageal squamous cell carcinoma risk in Taiwan: a case control study

**DOI:** 10.1186/1471-230X-9-37

**Published:** 2009-05-23

**Authors:** Huang-Ming Hu, Chao-Hung Kuo, Chien-Hung Lee, I-Chen Wu, Ka-Wo Lee, Jang-Ming Lee, Yih-Gang Goan, Shah-Hwa Chou, Ein-Long Kao, Ming-Tsang Wu, Deng-Chyang Wu

**Affiliations:** 1 Division of Internal Medicine, Kaohsiung Municipal Hsiao-Kang Hospital, Kaohsiung, Taiwan; 2 Division of Gastroenterology, Department of Internal Medicine, Kaohsiung Medical University Hospital, Kaohsiung, Taiwan; 3 Department of Medicine, Faculty of Medicine, College of Medicine, Kaohsiung Medical University, Taiwan; 4 Center of Excellence for Environmental Medicine, Kaohsiung Medical University, Kaohsiung, Taiwan; 5 Department of Public Health, Kaohsiung Medical University, Kaohsiung, Taiwan; 6 Department of Otolaryngology, Kaohsiung Medical University Hospital, Kaohsiung, Taiwan; 7 Department of Otolaryngology, Faculty of Medicine, College of Medicine, Kaohsiung Medical University, Taiwan; 8 Department of Surgery, National Taiwan University Hospital, Taipei, Taiwan; 9 Department of Chest Surgery, Kaohsiung Veterans General Hospital, Kaohsiung, Taiwan; 10 Department of Chest Surgery, Kaohsiung Medical University Hospital, Kaohsiung, Taiwan; 11 Department of Family Medicine, Graduate Institute of Occupational Safety and Health, Kaohsiung Medical University, Kaohsiung, Taiwan

## Abstract

**Background:**

Overexpression of Cyclooxygenase-2 (COX-2) was observed in many types of cancers, including esophageal squamous cell carcinoma (ESCC). One functional SNP, COX-2 -1195G/A, has been reported to mediate susceptibility of ESCC in Chinese populations. In our previous study, the presence of *Helicobacter pylori *(*H. pylori*) was found to play a protective role in development of ESCC. The interaction of COX-2 and *H. pylori *in gastric cancer was well investigated. However, literature on their interaction in ESCC risk is scarce. The purpose of this study was to evaluate the association and interaction between COX-2 single nucleotide polymorphism (SNP), *H. pylori *infection and the risk of developing ESCC.

**Methods:**

One hundred and eighty patients with ESCC and 194 controls were enrolled in this study. Personal data regarding related risk factors, including alcohol consumption, smoking habits and betel quid chewing, were collected via questionnaire. Genotypes of the COX-2 -1195 polymorphism were determined by PCR-based restriction fragment length polymorphism. *H. pylori *seropositivity was defined by immunochromatographic screening test. Data was analyzed by chi-squared tests and polytomous logistics regression.

**Results:**

In analysis adjusting for the covariates and confounders, *H. pylori *seropositivity was found to be inversely association with the ESCC development (adjusted OR: 0.5, 95% CI: 0.3 – 0.9). COX-2 -1195 AA homozygous was associated with an increased risk of contracting ESCC in comparison with the non-AA group, especially among patients with *H. pylori *seronegative (adjusted OR ratio: 2.9, 95% CI: 1.2 – 7.3). The effect was strengthened among patients with lower third ESCC (adjusted OR ratio: 6.9, 95% CI 2.1 – 22.5). Besides, *H. pylori *seropositivity conveyed a notably inverse effect among patients with COX-2 AA polymorphism (AOR ratio: 0.3, 95% CI: 0.1 – 0.9), and the effect was observed to be enhanced for the lower third ESCC patients (AOR ratio: 0.09, 95% CI: 0.02 – 0.47, *p *for multiplicative interaction 0.008)

**Conclusion:**

*H. pylori *seropositivity is inversely associated with the risk of ESCC in Taiwan, and COX-2 -1195 polymorphism plays a role in modifying the influence between *H. pylori *and ESCC, especially in lower third esophagus.

## Background

Esophageal cancer occurs worldwide with a variable geographic distribution[1] and the incidence is high in certain parts of China [2,3]. This malignancy has two histological subtypes: squamous cell carcinoma (ESCC) and adenocarcinoma. Most of the esophageal cancers are ESCC, although the incidence of the adenocarcinoma is increasing in West countries [4,5]. ESCC is one of the most fatal forms of carcinoma. Because of dissatisfying the improvements in prognosis, primary prevention and intervention are important in the control of the disease. The development of ESCC of the esophagus is a multifactor process associated with a variety of risk factors. Several environmental factors have been implicated in the pathogenesis of ESCC, including tobacco smoking, and alcohol drinking [6-9]. Recent research in Taiwan also mentioned that betel quid chewing was another important factor in developing ESCC [10,11]. However, some cases still developed ESCC without such risk factors, indicating that there are other risk factors associated with developing ESCC.

Overexpression of Cyclooxygenase (COX)-2 is observed in many types of cancers, including ESCC [12-14]. COX-2 is the inducible form of the enzyme for prostanoid synthesis, and the active products, such as prostaglandins and prostacyclin, have been implicated in carcinogenesis [15,16]. COX-2 is also involved in many processes fundamental to tumor development, such as apoptosis, cell adhesion, proliferation, invasion, metastasis and angiogenesis [17-19]. The COX-2 expression can be induced by variable stimuli, including cytokines and growth factors. But it is believed that transcription regulation is the major process in regulating the express of COX-2. Several naturally occurring single nucleotide polymorphism (SNP) in the COX-2 promoter region were observed and its distribution varied in different ethnics. One functional SNP, COX-2 -1195G/A, has been reported in Chinese populations previously and it locates in the core recognition sequence of c-MYB in the promoter region [20]. c-MYB is one of the nuclear proteins and it may have the ability to induce the transcription of COX-2 gene [20,21], and then to inhibit apoptosis by overexpression of COX-2 [17,21]. The SNP on c-MYB-recognized region can influence the expression of COX-2 and may play a role in mediating susceptibility of ESCC [20].

The role of *H. pylori *in development of ESCC is still puzzling. Several studies revealed the positive correlation between *H. pylori *infection and ESCC by histological pattern. An investigation from a Swedish population disclosed a positive association between ESCC and both *H. pylori Cag-A *positive infection and atrophic gastritis[22]. Bahmanyar *et al *[23] provided another finding that gastric ulcer patients had an 80% increased risk of ESCC, and supposed that corpus atrophy may play a role in ESCC etiology. On the contrary, *H. pylori *infection had also been mentioned to be associated with a decreased risk of developing ESCC [24,25]. According to the study from Wu *et al *[25], the protective effect of *H. pylori *infection was stronger in younger subjects, nonsmokers, nondrinkers and in the lower third cases of ESCC. This finding provides a clue that the influence of *H. pylori *infection in developing ESCC may vary according to the location in the esophagus.

Both overexpression of COX-2 and *H. pylori *infection were associated with the development of gastric adenocarcinoma [26-28]. The presence of *H. pylori *has also played a role in induction of expression of COX-2 in stomach [29-31]. However, the interaction of *H. pylori *and COX-2 in risk of ESCC has not been well investigated. In the present study, we conducted an incident case-control study to evaluate the relationship of SNP in the COX-2 promoter, *H. pylori *infection, and their interaction in the risk of developing ESCC.

## Methods

### Participants

All subjects were consecutively recruited at the Kaohsiung Medical University Hospital. This study was carried out according to the principles of the Declaration of Helsinki and was approved by the Institutional Review Board (IRB) of Kaohsiung Medical University Hospital (KMUH-IRB-960114). One hundred and eighty patients of ESCC and 194 controls were enrolled in this study. All patients of ESCC were histopathologically confirmed using gastroscopic biopsies. Their data were collected within one month after diagnosis, at the time when they admitted to hospital for staging or treatment. The controls were healthy and cancer-free individuals collected during the same period from the group receiving routine health examination, including gastroscopy at the same hospital. They were selected according the frequency matched to the patients based on age, sex and ethnicity. The exclusion criteria of both groups included previous cancer history, previous peptic ulcer history, previous *H. pylori *eradication history, and blood transfusion within 6 months.

Informed consent was obtained from each subject, and personal data regarding demographic characteristics such as sex, age and related risk factors, including alcohol consumption, smoking habits and betel quid chewing, were collected via questionnaire from each participant after interviewing with trained interviewer. Alcoholic drinker was defined if the subject had drunk wine, beer or distilled spirits more than once per week for at least 6 months. Those who had smoked more than 10 cigarettes per week for at least 6 months were defined as cigarette smokers. Those who chewed betel quid each week for at least the same period were defined as betel quid chewers, and we defined 10 betel quids as one pack.

### Location of ESCC

Location of the ESCC was identified by endoscopic finding or by the image of the chest computed tomography if the esophageal lumen containing ESCC was narrowing. Lesions were classified according to their location in upper, middle or lower third esophagus [32]. Upper third esophagus extended from cricopharnygeal sphincter (15 cm) to the tracheal bifurcation (23 cm). Lower third esophagus extended from the approximate level of T9 vertebral body (32 cm) to the gastroesophageal junction (40 cm). If the lesion involved more than one region, location was defined according the high percentage of mass located.

### COX-2 -1195 polymorphism detection

Peripheral blood samples were collected from all patients and controls. Heparinized whole blood was centrifuged at 2,000 rpm for 10 min to isolate plasma supernatant (for *H. pylori *detection, described as below). Genomic DNA was extracted from remained blood cells using standard phenol-chloroform method. Genotypes of the COX-2 promoter region containing -1195G → A were determined by PCR-based restriction fragment length polymorphism (PCR-RFLP). The PCR primer pairs used to amplify the COX-2 promoter region were 5'-CCC TGA GCA CTA CCC ATG AT-3' (forward) and 5'-GCC CTT CAT AGG AGA TAC TGG-3' (reverse). PCR was performed at a 25 μL reaction mixture containing 1.5 μl of DNA template, 1 unit of Taq DNA polymerase (Promega, Madison, WI), 0.1 μmol/L of each primer, 0.2 mmol/L of deoxynucleoside triphosphate, 1.5 mmol/L of MgCl_2_, and 1× reaction buffer. The PCR profile included an initial melting step of 2 minutes at 95°C, followed by 35 cycles of 30 seconds at 95°C, 30 seconds at 60°C, 45 seconds at 72°C, and a final elongation step of 5 minutes at 72°C. Restriction enzymes PvuII (New England Biolabs, Beverly, MA) was used to distinguish -1195G → A genotype. Genotyping was performed without knowledge of the case or control status of the subjects.

### *H. pylori *detection

Because of the variable stenotic change of esophagus in ESCC subjects, we could not well evaluate the *H. pylori *infection status by sampling from gastric tissue. So *H. pylori *seropositivity was defined according to the existence of IgG antibody in plasma. The isolated plasma was stored at -70°C until analyzed. A commercial immunochromatographic screening test (Chimbio *H. pylori *STAT-PAK, Chimbio Diagnostic System, Inc., Medford NY) was used to detect *H. pylori *antibodies in plasma. The results were read 10 minutes by two research technicians after the subject's plasma was placed into the sample well. In accordance with the manufacturer's instructions, the sensitivity and specificity of the immunochromatographic test were 94% and 98% respectively.

### Statistic analysis

Univariate analysis was performed with the chi-squared tests in order to search for associations between demographic characteristics and ESCC. The Hardy-Weinberg equilibrium test was used to assess the discrepancies between the observed and the expected COX-2 1195G → A genotype frequencies among control subjects. Because of two subgroups of ESCC cases were defined (upper two third and lower third), polytomous logistics regression was applied in the multivariate analysis to determine the risk of contracting the two defined cancer groups [33,34]. Estimates were adjusted for covariates and confounders, including age, gender, educational level and consumption of tobacco, alcohol and betel quid, in the regression models. The potential interaction between COX-2 1195G → A genotype and *H. pylori *seropositivity in the development of ESCC was evaluated by fitting a multiplicative model with cross-product terms representing the interaction. Two-sided P-values of 0.05 or below were considered statistically significant. All analyses were conducted using the statistical packages of Stata[35].

## Results

The demographic characteristics and substance use in both ESCC patients and controls were listed in Table 1. The majority of ESCC patients were male. Substantial differences in the distribution with regard to education level, tobacco smoking, alcohol drinking and betel quid chewing were found between ESCC and controls. After adjusting for covariates, high education level (>12 years) had a significantly reduced risk (adjusted odds ratio (AOR): 0.2, 95% CI: 0.1 – 0.6) of developing ESCC. On the contrary, tobacco smoking, alcohol drinking and betel quid chewing were associated with an increased risk of ESCC development, with a clear dose-dependent effect in the latter two substances.

**Table 1:** Distributions and odds ratios of ESCC associated with selected demographic characteristics and substance uses

Factor/Category	Cases	Controls	
		
	No. (%)	No. (%)	AOR^1 ^(95% CI)
Total No.	180	194	
Anatomic subsite of the esophagus			
Upper 1/3	39 (22)		
Middle 1/3	83 (46)		
Lower 1/3	58 (32)		
Age (years)			
<50	63 (35)	70 (36)	1.0
51–60	36 (20)	58 (30)	0.7 (0.4 – 1.2)
61–70	45 (25)	36 (18)	1.4 (0.8 – 2.4)
>70	36 (20)	30 (16)	1.3 (0.7 – 2.4)
Gender			
Female	5 (3)	7 (4)	1.0
Male	175 (97)	187 (96)	1.3 (0.4 – 4.2)
Ethnicity			
Fukienese	138 (77)	152 (78)	1.0
Aborigines	31 (17)	28 (15)	1.2 (0.7 – 2.1)
Others	11 (6)	14 (7)	0.9 (0.4 – 2.0)
Education level (years)			
<7	94 (52)	51 (26)	1.0
7–12	77 (43)	86 (44)	0.7 (0.4 – 1.4)
>12	9 (5)	57 (30)	0.2 (0.1 – 0.6)
*p *for trend			0.001
Tobacco smoking (pack-years)^2^			
No	14 (8)	101 (52)	1.0
1–20	37 (20)	26 (13)	8.7 (3.2 – 23.6)
>20	129 (72)	67 (35)	7.7 (3.4 – 17.7)
*p *for trend			<0.001
Alcohol drinking (drink-years)^2^			
No	36 (20)	130 (67)	1.0
1–40	44 (24)	34 (18)	3.0 (1.4 – 6.4)
>40	100 (56)	30 (15)	7.2 (3.6 – 14.7)
*p *for trend			<0.001
Betel quid chewing (pack-years)^2^			
No	82 (46)	175 (90)	1.0
1–20	26 (15)	7 (4)	3.9 (1.4 – 10.8)
>20	69 (39)	12 (6)	5.6 (2.5 – 12.5)
*p *for trend			<0.001

^1 ^Odds ratios were adjusted for covariates in the table.

^2^One drink corresponds to 15.75-g of alcohol; one smoked pack and chewed pack correspond to 20-cigarettes and 10-betel quids, respectively.

The protective effect of *H. pylori *infection on ESCC was assessed in Table 2. Sixty two of 180 (37%) ESCC patients and 102 of 194 (53%) controls were *H. pylori *seropositive. After controlling for the covariates, *H. pylori *seropositivity was found to confer a 0.5-fold risk (95% CI: 0.3 – 0.9) of developing ESCC. To further evaluate the effect of *H. pylori *infection on the ESCC location, ESCC cases were classified as two subgroups: lower third and upper two thirds. Compared with the controls (53%), the lower third (34%) and upper two thirds (38%) of ESCC patients had a lower positive response for *H. pylori*, showing a possible protective effect in the risk of developing ESCC.

**Table 2:** Adjusted OR for cancers at upper, middle and lower third of the esophagus associated with seropositivity of *H. pylori*

*H. pylori *infection	Controls	Location of ESCC				Total cases	
				
		Upper + Middle		lower			
	
	No. (%)	No. (%)	AOR^1^ (95% CI)	No. (%)	AOR^1^ (95% CI)	No. (%)	AOR^1^ (95% CI)
Negative	92 (47)	76 (62)	1.0	38 (66)	1.0	114 (63)	1.0
Positive	102 (53)	46 (38)	0.6 (0.3 – 1.1)	20 (34)	0.5 (0.2 – 1.0)	66 (37)	0.5 (0.3 – 0.9)

^1 ^Odds ratios were adjusted for the covariates (age, gender, education level, pack-years of cigarette smoking and of betel quid chewing, and drink-years of alcohol drinking) listed in Table 1.

The distribution of COX-2 -1195 polymorphism both for the cases and controls was shown in Table 3. The allelic frequency for -1195G was 0.52 in controls and 0.44 in ESCC patients. The polymorphic distribution for COX-2 -1195 gene was found to well comply with the Hardy-Weinberg equilibrium among the controls (P > 0.05). Multivariate polytomous logistic regression analyses showed that COX-2 -1195AA homozygous was associated with an increased risk of contracting ESCC (AOR: 2.3, 95% CI: 1.0 – 5.2). Compared with the non-AA subgroup, a 2.5-fold significantly elevated risk (95% CI: 1.0 – 6.1) was identified for lower third ESCC patients who carried the AA homozygote.

**Table 3:** Adjusted OR for cancers at upper, middle and lower third of the esophagus associated with COX-2 -1195G/A polymorphism.

COX-2 polymorphism	Controls	Location of ESCC				Total cases	
				
		Upper + Middle		Lower			
	
	No. (%)	No. (%).	AOR^1^ (95% CI)	No. (%).	AOR^1^ (95% CI)	No. (%).	AOR^1^ (95% CI)
GG	50 (26)	23 (19)	1.0	16 (28)	1.0	39 (22)	1.0
AG	103 (53)	59 (48)	1.7 (0.7 – 4.0)	21 (36)	0.9 (0.3 – 2.3)	80 (44)	1.2 (0.6 – 2.4)
AA	41 (21)	40 (33)	2.0 (0.7 – 5.2)	21 (36)	2.3 (0.8 – 6.7)	61 (34)	2.3 (1.0 – 5.2)
*p *for H-W^2^	0.471						
							
GG+AG	153 (79)	82 (67)	1.0	37 (64)	1.0	119 (66)	1.0
AA	41 (21)	40 (33)	1.4 (0.7 – 2.9)	21 (36)	2.5 (1.0 – 6.1)	61 (34)	2.0 (1.1 – 3.9)

^1 ^Odds ratios were adjusted for the covariates (age, gender, education level, pack-years of cigarette smoking and of betel quid chewing, and drink-years of alcohol drinking) listed in Table 1.

^2^*p *value for Hardy-Weinberg equilibrium test.

To understand the influence of COX-2 polymorphism on the ESCC risk with regard to the status of *H. pylori *infection, we presented effect modification stratification analysis in Table 4. Among *H. pylori *seropositive patients, no significant relationship between COX-2 -1195 genotypes and ESCC was detected. However, among *H. pylori *seronegative subjects, -1195AA homozygous was found to confer an increased risk in developing ESCC in comparison with non-AA polymorphisms (AOR ratio: 2.9, 95% CI: 1.2 – 7.3). This effect was strengthened among patients with lower third ESCC (AOR ratio: 6.9, 95% CI: 2.1 – 22.5). Alternatively, *H. pylori *seropositivity conveyed a notably inverse effect among patients with COX-2 AA polymorphism (AOR ratio: 0.3, 95% CI: 0.1 – 0.9). Similarly, such opposite effect was observed to be enhanced for the lower third ESCC patients (AOR ratio: 0.09, 95% CI: 0.02 – 0.47). Compared with that for COX-2 non-AA carriers (AOR ratio 1.0), significantly heterogeneous inverse cancer risk for AA carriers was identified at this subsite of ESCC (*p *for multiplicative interaction 0.008), though limited study samples were used to assess the effect.

**Table 4:** The effect modification between COX-2 -1195G/A polymorphism and seropositivity of *H. pylori *on the risk of ESCC at lower third of the esophagus and all cancers

COX-2 polymorphism	Lower third ESCC cases					Total ESCC cases				
		
	*H. pylori*-negative		*H. pylori*-positive		*H. pylori *(+) vs. (-) OR ratio (95% CI)	*H. pylori*-negative		*H. pylori*-positive		*H. pylori *(+) vs. (-) OR ratio (95% CI)
				
	Cases/Controls	AOR^1 ^(95% CI)	Cases/Controls	AOR^1 ^(95% CI)		Cases/Controls	AOR^1 ^(95% CI)	Cases/Controls	AOR^1 ^(95% CI)	
GG+AG	20/72	1.0	17/81	1.0 (0.4 – 2.5)	1.0 (0.4 – 2.5)	73/72	1.0	46/81	0.7 (0.4 – 1.4)	0.7 (0.4 – 1.4)
AA	18/20	6.9 (2.1 – 22.5)	3/21	0.6 (0.1 – 2.8)	0.09 (0.02 – 0.47)	41/20	2.9 (1.2 – 7.3)	20/21	0.9 (0.4 – 2.1)	0.3 (0.1 – 0.9)
AA vs. GG+AG OR ratio (95% CI)		6.9 (2.1 – 22.5)		0.6 (0.1 – 2.7)			2.9 (1.2 – 7.3)		1.2 (0.5 to 3.0)	
*p *for interaction^2^				0.008					0.172	

^1 ^Odds ratios were adjusted for the covariates (age, gender, education level, pack-years of cigarette smoking and of betel quid chewing, and drink-years of alcohol drinking) listed in Table 1.

^2^Interaction was examined with likelihood ratio test for cross-product terms based on a multiplicative model.

## Discussion

Several studies have suggested the association between overexpression of COX-2 and ESCC [12-14]. According the previous study, COX-2 -1195G/A polymorphism, a functional SNP disclosed in Chinese populations, could modified not only COX-2 mRNA level, but the risk of ESCC[20]. In the present study, we found similar result that COX-2 -1195AA homozygous, which had higher COX-2 mRNA level, was related to increased risk of ESCC (AOR: 2.0, 95% CI: 1.1 – 3.9) compared with non-AA genotype subjects in multivariate logistic regression analysis. The trend was the same in the lower third ESCC cases (AOR: 2.5, 95% CI: 1.0 – 6.1). However, the relationship was absent in upper two third esophageal area. This is consistent with the existing evidences from Kawabe *et al *[36] and Sivula *et al *[37], which mentioned that high COX-2 expression in ESCC was found significantly more often in lower parts of the esophagus. These results suggest that the increased risk of ESCC in the lower third part of the esophagus may be associated with the COX-2 -1195AA homozygous, which induces higher COX-2 expression.

In our previous study as Wu *et al *[25], we mentioned that subjects with positive *H. pylori *infection had reduced risk of developing ESCC. In the present study with newly collected ESCC subjects and controls, we also found the same effect of *H. pylori *in ESCC. On the contrary, there are different findings. Bahmanyar *et al*[23] found that patients with gastric ulcer history had higher risk of developing ESCC, with supposed positive *H. pylori *infection status and corpus atrophy change via ulcer presentation. We did not evaluate the association between gastric ulcer and ESCC because a higher proportion of gastric ulcer was induced by drugs in Taiwan [38], especially in elderly subjects who were also the high risk group of ESCC by age.

The COX-2 expression induced by *H. pylori *infection has been well evaluated in gastric mucosa previously [29,39], but research about the interaction in esophageal squamous cells is scarce. A study concerning the histological change of low esophageal mucosa before and after *H. pylori *eradication disclosed that COX-2 expression gradually increased after treatment [40]. This observation suggests that COX-2 expression in low esophageal region is lower under *H. pylori *seropositive status. In the present study, we put the COX-2 -1195G/A polymorphism under consideration and found that COX-2 -1195AA homozygote in *H pylori *seronegative status had increased ESCC risk (AOR ratio: 2.9, 95% CI: 1.2 – 7.3). This effect was strengthened among subjects with lower third ESCC (AOR ratio: 6.9, 95% CI 2.1 – 22.5). Furthermore, *H. pylori *seropositivity conveyed a notably inverse effect in ESCC risk (AOR ratio: 0.3, 95% CI: 0.1 – 0.9) in subjects carrying COX-2 AA genotype. Such opposite effect was observed to be enhanced for the lower third ESCC patients (AOR ratio: 0.09, 95% CI: 0.02 – 0.47). However, such relationship between *H. pylori *infection and the risk of ESCC did not persist in COX-2 non-AA genotype. These findings suggest that the protective effect to lower third ESCC risk provided by *H. pylori *seropositivity may be mediated by influencing COX-2 AA genotype expression. Our data also indicated the area in which to search for underlying mechanism.

In consideration of the lower third part of the esophagus, one of the possible changes under chronic *H. pylori *infection is the reduced load of esophageal acid. Long-term *H. pylori *infection may provoke gastric atrophy, accompany with hypochlorhydria [41-44], and the prevalence increased with advancing age [45]. According to the study from Lurje *et al *[46], COX-2 mRNA expression is significantly increased in acid-exposed compared to non-exposed squamous epithelium. Another study from Vallbohmer *et al *[47] disclosed that COX-2 gene expression was increased in the distal esophageal squamous mucosa of most patients with gastro-esophageal reflux disease, and the increased COX-2 expression was usually normalized following anti-reflux surgery. These findings provide a clue that ESCC development in the lower part of the esophagus may have a similar carcinogenesis pathway as esophageal adenocarcinoma in consideration of acid exposure risk. However, further studies are needed to evaluate the relationship between acid exposure in squamous cell epithelium and development of ESCC.

There are some limitations to the present study. Firstly, we selected controls only from the group receiving health examination in which gastroscopy was included. Selection bias resulting from the will of receiving invasive gastroscopy might have distorted our findings. However, the distribution of COX-2 -1195 genotypes in controls still complies the Hardy-Weinberg equilibrium. In addition, the *H. pylori *infection status was determined according to the result of serologic method. The ESCC location was determined relying on the image of chest computed tomography in part of ESCC cases. Misclassification happened in these variables might influence our study results. Furthermore, limited studied samples might lead to a relatively lower statistical power in certain subgroups. On the other hand, the strength of our study is that previous studies [11,34,48] for the relationship between habitual substance use and the risk of ESCC from the same area of Taiwan provide us adequate information to well design the questionnaire and to adjust the influence from those potential confounding factors.

## Conclusion

This study provided evidence that both COX-2 -1195G/A polymorphism and *H. pylori *infection had influence in risk of ESCC in Taiwanese population. Subjects carrying COX-2 -1195 AA homozygote has increased risk of ESCC in lower third esophagus. The *H. pylori *seropositivity had an inverse association in ESCC development, and it was observed to be enhanced in subgroup of COX-2 -1195AA genotype, especially when ESCC is located in lower third esophagus. These findings suggest that COX-2 -1195 polymorphism plays a role in modifying the inverse association between *H. pylori *infection and risk of ESCC. The underlying mechanism needs further investigation.

## Abbreviations

ESCC: esophageal squamous cell carcinoma; *COX-2*: *cyclooxygenase 2*; SNP: single nucleotide polymorphism; *H. pylori*: *helicobacter pylori*; PCR: polymerase chain reaction; RFLP: restriction fragment length polymorphism; AOR: adjusted odds ratio; CI: confidence interval.

## Competing interests

The authors declare that they have no competing interests.

## Authors' contributions

HMH participated in the design of the study, carried out the genetic analysis and drafted the manuscript. CHK performed literature review and administered to the direction of discussion section. CHL and MTW participated in the design of the questionnaire and performed the statistic analysis. ICW carried out the immunochromatographic test and participated in collections of clinical data. JML and YGG participated in study design. KWL, SHC and ELK assisted in the collection of clinical data. DCW participated in its design and coordination, and supervised the study. All authors read and approved the final manuscript.

## Pre-publication history

The pre-publication history for this paper can be accessed here:

http://www.biomedcentral.com/1471-230X/9/37/prepub
